# Risk factor analysis and prediction model construction for contralateral central lymph node metastasis in unilateral papillary thyroid carcinoma: Sex, extrathyroidal tumor extension (ETE), tumor diameter and ipsilateral central lymph node metastasis (ICLNM) are risk factor for unilateral papillary thyroid carcinoma (UPTC): a case control study

**DOI:** 10.1186/s12957-024-03565-5

**Published:** 2024-10-25

**Authors:** Jihao Qin, Xiaowen Fang, Chenxi Liang, Siyu Li, Xueyu Zeng, Hancheng Jiang, Zhu Chen, Jie-Hua Li

**Affiliations:** 1 Department of Gastrointestinal and Gland Surgery, The First Affiliated Hospital of Guangxi Medical University, No. 6 Shuangyong Road, Nanning, 530021 China; 2 Department of Thyroid Surgery, the Fourth Affiliated Hospital of Guangxi Medical University, Liuzhou, 545007 China

**Keywords:** Thyroid cancer, Unilateral papillary thyroid carcinoma, Contralateral central lymph node metastasis, Nomogram

## Abstract

**Objective:**

To investigate contralateral central lymph node metastasis (CCLNM) in patients with unilateral papillary thyroid carcinoma (UPTC). To provide a reference for clinical decision-making, a prediction model for the probability of CCLNM was established.

**Method:**

The clinicopathological data of 221 UPTC patients who underwent surgical treatment were retrospectively analyzed. Univariate and multivariate logistic regression analyses were performed to determine the independent risk factors for CCLNM according to clinicopathological characteristics, construct a prediction model to construct a visual nomogram, and evaluate the model.

**Results:**

According to univariate and multivariate logistic regression analyses, sex (*P* = 0.01, OR: 3.790, 95% CI: 1.373–10.465), extrathyroidal tumor extension (ETE) (*P* = 0.040, OR: 6.364, 95% CI: 1.083–37.381), tumor diameter (*P* = 0.010, OR: 3.674, 95% CI: 1.372–9.839) and ipsilateral central lymph node metastasis (ICLNM) (*P* < 0.001, OR: 38.552, 95% CI: 2.675–27.342) were found to be independent risk factors for CCLNM and were used to construct a nomogram for internal verification. The ROC curve had an AUC of 0.852 in the training group and an AUC of 0.848 in the verification group, and the calibration curve indicated that the prediction probability of the model was consistent with the actual probability. Finally, the analysis of the decision curve showed that the model has good application value in clinical decision-making.

**Conclusion:**

Sex, ETE, tumor size, and ICLNM emerged as independent risk factors for CCLNM in UPTC patients. A predictive model was therefore developed, harnessing these variables to enable an objective, personalized estimation of CCLNM risk. This tool offers valuable insights to inform surgical planning and optimize treatment strategies for UPTC management.

**Supplementary Information:**

The online version contains supplementary material available at 10.1186/s12957-024-03565-5.

## Introduction

The global incidence of thyroid cancer (TC) has markedly increased in recent years, predominantly attributable to the increase in papillary thyroid carcinoma (PTC), which constitutes approximately 90% of all TC cases [1]. Notably, PTC frequently undergoes lymphatic spread to the central lymph nodes at the initial stage. Cervical lymph node metastasis significantly exacerbates the risk of recurrence and adversely affects survival rates in TC patients [2–4]. Moreover, identifying central lymph node metastasis can help determine the tumor lymph node metastasis stage (TNM), assess the need for thyroid stimulating hormone (TSH) suppression therapy or radioiodine (RAI) therapy, and predict the likelihood of lateral cervical lymph node metastasis (LLNM).

In managing PTC, the addition of bilateral central lymph node dissection (BCLND) to total thyroidectomy (TT) has the potential to lower local recurrence rates, particularly in mitigating central recurrence [5]. Despite this benefit, the early-stage predilection of PTC for lymph node metastasis, alongside the technical complexity of cervical lymph node dissection, results in substantial increases in the risk of postoperative complications, including impairments to parathyroid function and damage to the recurrent laryngeal nerve and surrounding vasculature [6], associated with BCLND. Moreover, the sensitivity and specificity for evaluating CLNM in PTC patients were 67.7% and 83.1%, respectively [7]. Ultrasound-guided thyroid fine needle aspiration biopsy (US-FNAB), an invasive examination, is not routinely used to evaluate cervical lymph node metastasis before surgery. In conclusion, the preoperative ultrasonographic detection of CLNM has limited diagnostic sensitivity and specificity, at only 50-70% [8], and there is still a lack of objective quantitative methods to accurately determine the presence of CCLNM metastasis before surgery.

This retrospective study examined the clinical and pathological data of UPTC patients who were treated with total thyroidectomy in conjunction with BCLND. The objective of this study was to identify risk factors for CCLNM in UPTC patients and to devise a prognostic model. Such a model aims to optimize treatment outcomes while simultaneously reducing the frequency of surgical complications during clinical diagnosis and management.

## Materials and methods

### Research subjects

Patients with PTC who underwent surgical treatment at the First, Fourth and Fifth Affiliated Hospitals of Guangxi Medical University from January 2020 to June 2023 were screened and included in this study. The inclusion criteria for patients were as follows: (1) had traceable and complete clinicopathological data; (2) underwent surgery including total thyroidectomy (TT) + BCLND; (3) underwent a primary operation; and (4) had a final pathological type of unilateral glandular PTC. The exclusion criteria for patients were as follows: (1) had previously undergone thyroid surgery/ablation; (2) had a history or coexistence of other head and neck malignancies; (3) had a thyroid isthmus tumor; and (4) had incomplete clinicopathological data. A total of 221 UPTC patients were enrolled in the study and assigned to the training or validation group at a 7:3 ratio using the random sequence method. According to the pathological CCLNM metastasis results, the patients were divided into a CCLNM-negative group and a CCLNM-positive group.

### Data source

Clinical data collected included age, sex, body mass index (BMI), TSH, Hashimoto’s thyroiditis (HT), tumor diameter, capsular invasion, ETE, vascular cancer thrombus, multifocality, ICLNM, CCLNM and other clinicopathologic data.

### Statistical processing

SPSS 27.0 was used for the statistical analysis of all the data, and a normality test was conducted for the measurement data. Normally distributed data are represented as the mean ± standard deviation, and a t test was used for comparisons between groups. Those who did not fit the normal distribution were described by the median and interquartile range (IQR), and comparisons between groups were nonparametric. Count data are presented as frequencies (percentages). The chi-square (X2) test was used for comparisons between groups, and *P* < 0.05 was considered to indicate statistical significance. Binary logistic multivariate regression analysis with t tests and X2 tests was used to analyze the risk factors identified by univariate regression analysis.

### Model establishment and verification

RStudio 4.2.1 was used to build a model and construct a visual nomogram. The established nomogram model of CCLNM in UPTC patients was internally validated with data from the validation cohort. Model differentiation was evaluated by the area under the receiver operating characteristic (ROC) curve (AUC). Model calibration was evaluated using the Hosmer–Leme test (goodness of fit test) in conjunction with a clinical decision curve analysis (DCA) to evaluate the clinical benefit of the model.

## Results

### Clinicopathological data of the enrolled patients

According to the specific inclusion and exclusion criteria, our study included 221 patients who were diagnosed with UPTC. The gender distribution included 50 males (22.6%), while females represented 161 individuals (77.4%). These patients had an average age of 37.7 years, with 150 patients being younger than 45 years and the remaining 71 being 45 years or older. According to the postoperative pathological data, the incidence of ICLNM was 58.8% (130/221), that of CCLNM was 35.3% (78/221), and that of bilateral central lymph node metastasis (BCLNM) was 33.0% (73/221). There was no metastasis to the ipsilateral central lymph nodes, while only 2.26% (5/221) of the patients had metastasis to the contralateral central lymph nodes (jump metastasis). The study cohort was further divided into a model group comprising 154 patients, 37.0% (57/154) of whom exhibited CCLNM, and the remaining patients did not. The internal validation group, comprising 67 patients, had a CCLNM incidence of 31.3% (21/67). The clinicopathological data for both the model group and the validation group are comprehensively illustrated in Table 1.

**Table 1:** Comparison of clinicopathologic-ictal data between the training group and verification group

Variables	Model group(*n* = 154)	Alidation group(*n* = 67)	*P* value
Sex [*n* (%)]			
Male	38(24.7)	12(17.9)	0.269
Female	116(75.3)	55(82.1)	
Age[years, *n*(%)]			
<45	103(66.9)	47(70.1)	0.633
≥ 45	51(33.1)	20(29.9)	
BMI(kg/m2)			
<25	107(69.5)	51(76.1)	0.315
≥ 25	47(30.5)	16(23.9)	
TSH[mIU/L, M, (QR)]	1.72(1.24,2.47)	1.99(1.08,2.43)	0.831
HT [*n* (%)]			
Yes	47(30.5)	28(41.8)	0.104
No	107(69.5)	39(58.2)	
Multifocality [*n* (%)]			
Yes	33(21.4)	9(13.4)	0.164
No	121(78.6)	58(86.6)	
Capsular invasion [*n* (%)]			
Yes	37(24.0)	18(26.9)	0.654
No	117(76.0)	49(73.1)	
ETE [*n* (%)]			
Yes	14(9.1)	5(7.5)	0.691
No	140(90.9)	62(92.5)	
Vascular cancer thrombus [*n* (%)]			
Yes	3(1.9)	4(6.0)	0.250
No	151(98.1)	63(94.0)	
Tumor diameter[cm, *n*(%)]			
<1 cm	69(44.8)	29(43.3)	0.834
≥ 1 cm	85(55.2)	38(56.7)	
ICLNM [*n* (%)]			
Yes	60(39.0)	31(46.3)	0.310
No	94(61.0)	36(53.7)	
CCLNM [*n* (%)]			
Yes	57(37.0)	21(31.3)	0.418
No	97(63.0)	46(68.7)	

BMI: Body mass index; TSH: Thyroid stimulating hormone; HT: Hashimoto’s thyroiditis; ETE: Extrathyroidal tumor extension; ICLNM: Ipsilateral central lymph node metastasis; CCLNM: Contralateral central lymph node metastasis

### Analysis of risk factors for CCLNM in the training group

Univariate analysis revealed that sex (*P* < 0.01), age (*P* = 0.016), capsular invasion (*P* = 0.015), extrathyroid invasion (*P* < 0.01), tumor diameter (*P* < 0.01), and ICLNM (*P* < 0.01) were significantly correlated with CCLNM (Table 2).

**Table 2:** Univariate analysis of CCLNM in training group

Variables	Negative(*n* = 97)	Positive(*n* = 57)	*P* value
Sex [*n* (%)]			
Male	15(15.5)	23(40.4)	<0.01
Female	82(84.5)	34(59.6)	
Age[years, *n*(%)]			
<45	58(59.8)	45(78.9)	0.016
≥ 45	39(40.2)	12(21.1)	
BMI(kg/m2)			
<25	69(71.1)	38(66.7)	0.561
≥ 25	28(28.9)	19(33.3)	
TSH[mIU/L, M, (QR)]	1.65(1.18,2.36)	1.89(1.48,2.60)	0.064
HT [*n* (%)]			
Yes	28(28.9)	19(33.3)	0.561
No	69(71.1)	38(66.7)	
Multifocality [*n* (%)]			
Yes	22(22.7)	11(19.3)	0.622
No	75(77.3)	46(80.7)	
Capsular invasion [*n* (%)]			
Yes	18(18.6)	20(35.1)	0.015
No	79(81.4)	37(64.9)	
ETE [*n* (%)]			
Yes	2(2.1)	12(21.1)	<0.01
No	95(97.9)	45(78.9)	
Vascular cancer thrombus [*n* (%)]			
Yes	0(0.0)	3(5.3)	0.999
No	97(100.0)	54(94.7)	
Tumor diameter[cm, *n*(%)]			
<1 cm	56(57.7)	13(22.8)	<0.01
≥ 1 cm	41(43.3)	44(77.2)	
ICLNM [*n* (%)]			
Yes	40(41.2)	53(93.0)	<0.01
No	57(58.8)	4(7.0)	

BMI: Body mass index; TSH: Thyroid stimulating hormone; ETE: Extrathyroidal tumor extension; ICLNM: Ipsilateral central lymph node metastasis;

Statistically significant indicators were selected from the above univariate analysis and included in multiple logistic regression analysis, and the results showed that sex (*P* = 0.01, OR: 3.790, 95% CI: 1.373–10.465), extrinsic thyroid invasion (*P* = 0.040, OR: 6.364, 95% CI: 1.083–37.381), tumor diameter (*P* = 0.010, OR: 3.674, 95% CI: 1.372–9.839) and ICLNM (*P* < 0.001, OR: 38.552, 95% CI: 2.675–27.342) were found to be independent risk factors for CCLNM (Table 3).

**Table 3:** Multifactor analysis of CCLNM in training group

Variables	β	*P*	OR(95 CI)
Sex	1.332	0.010	3.790(1.373–10.465)
Age	-0.905	0.069	0.405(0.153–1.072)
Capsular invasion	0.004	0.994	1.004(0.362–2.787)
ETE	1.851	0.040	6.364(1.083–37.381)
Tumor diameter	1.301	0.010	3.674(1.372–9.839)
ICLNM	2.146	<0.001	8.552(2.675–27.342)

ETE: Extrathyroidal tumor extension; ICLNM: Ipsilateral central lymph node metastasis

### Construction and verification of the CCLNM prediction model for UPTC patients

#### Construction of the prediction model

**Fig. 1:**
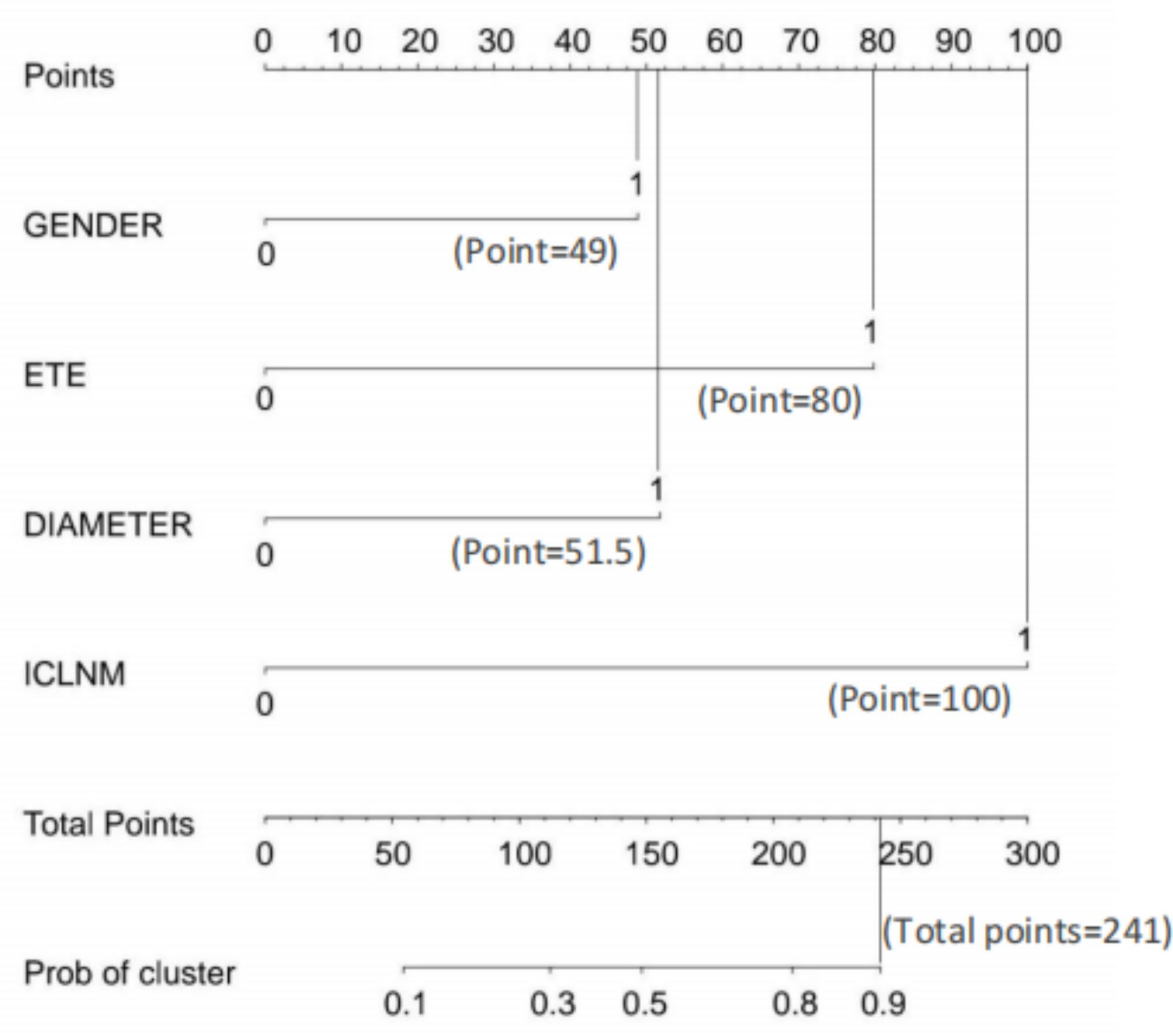
A nomogram for predicting CCLNM in UPTC patients. *Links to the online version: https://nomogramapp.shinyapps.io/DynNomapp/

Based on the independent risk factors screened by multifactor binary logistic regression (Table 3), a prediction model of CCLNM in UPTC patients was constructed using R language, and a nomogram was constructed, we also provide an online version of the prediction model at the following link (Fig. 1).

#### Prediction model validation

**Fig. 2:**
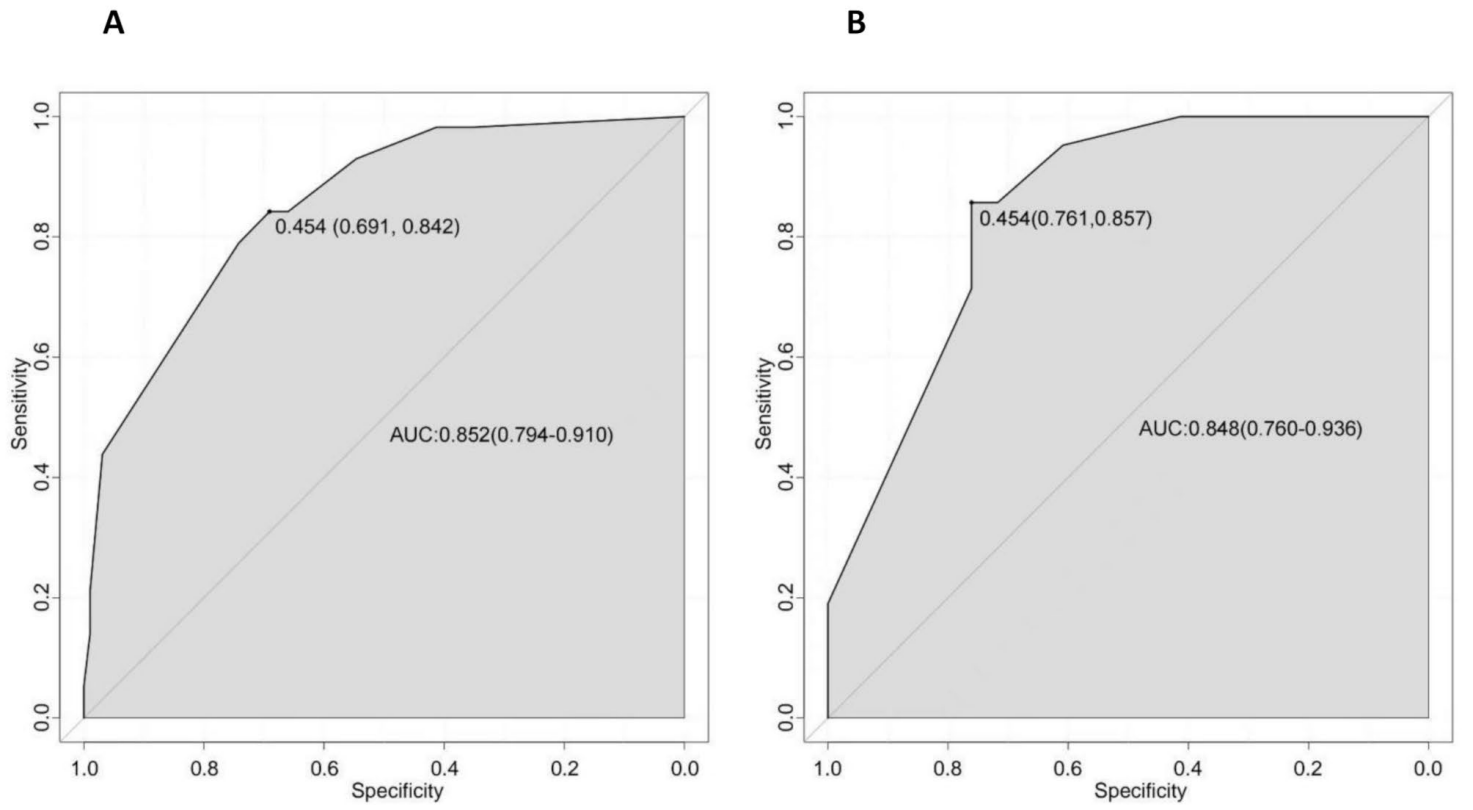
ROC curves of the training group and verification group. *A: ROC curves of the training group; **B**: ROC curves of the verification group

The ROC curve of the nomogram showed that the AUC of the training group was 0.852 (95% CI: 0.794 to 0.910), and the specificity and sensitivity of the training group were 69.1% and 84.2%, respectively, when the Yoden index was the highest. The AUC of the validation group was 0.848 (95% CI: 0.760–0.936). The specificity and sensitivity were 0.852 (95% CI: 0.794 to 0.910), respectively, when the Youden index was the highest. The nomogram had good discriminating powe, with true positive rate (TP) = 0.842, true negative rate (TN) = 0.691, false positive rate (FP) = 0.309 and false negative rate (FN) = 0.158 (Fig. 2).

**Fig. 3:**
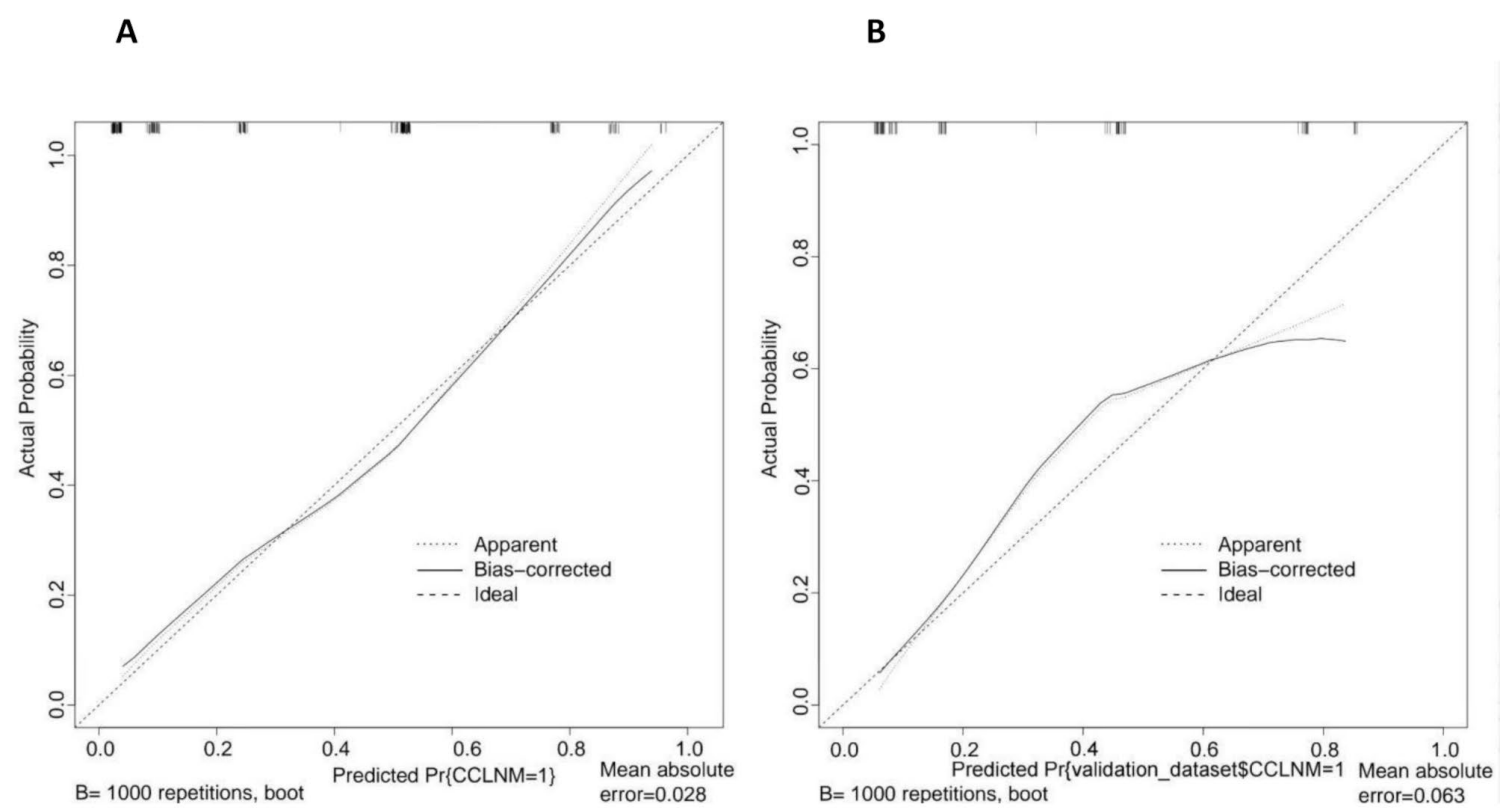
Calibration curves for the training group and validation group. *A: Calibration curves for the training group; **B**: Calibration curves for the validation group

A bootstrap approach was used to repeatedly sample the samples 1000 times for internal verification of the model. The probability of CCLNM of UPTC patients predicted by the column graph was plotted on the X-axis, while the actual probability was plotted on the Y-axis. The 45° line represents the ideal prediction curve of the model, and the solid line represents the actual prediction performance of the model. The average absolute error of the training group was 0.028, and the average absolute error of the verification group was 0.064, indicating that the predicted probability of the column graph was highly consistent with the actual probability (Fig. 3).

The P value of the Hosmer‒Lemeshow test (goodness of fit test) for this model was 0.922, which was greater than 0.05, indicating that the model had a good fit.

**Fig. 4:**
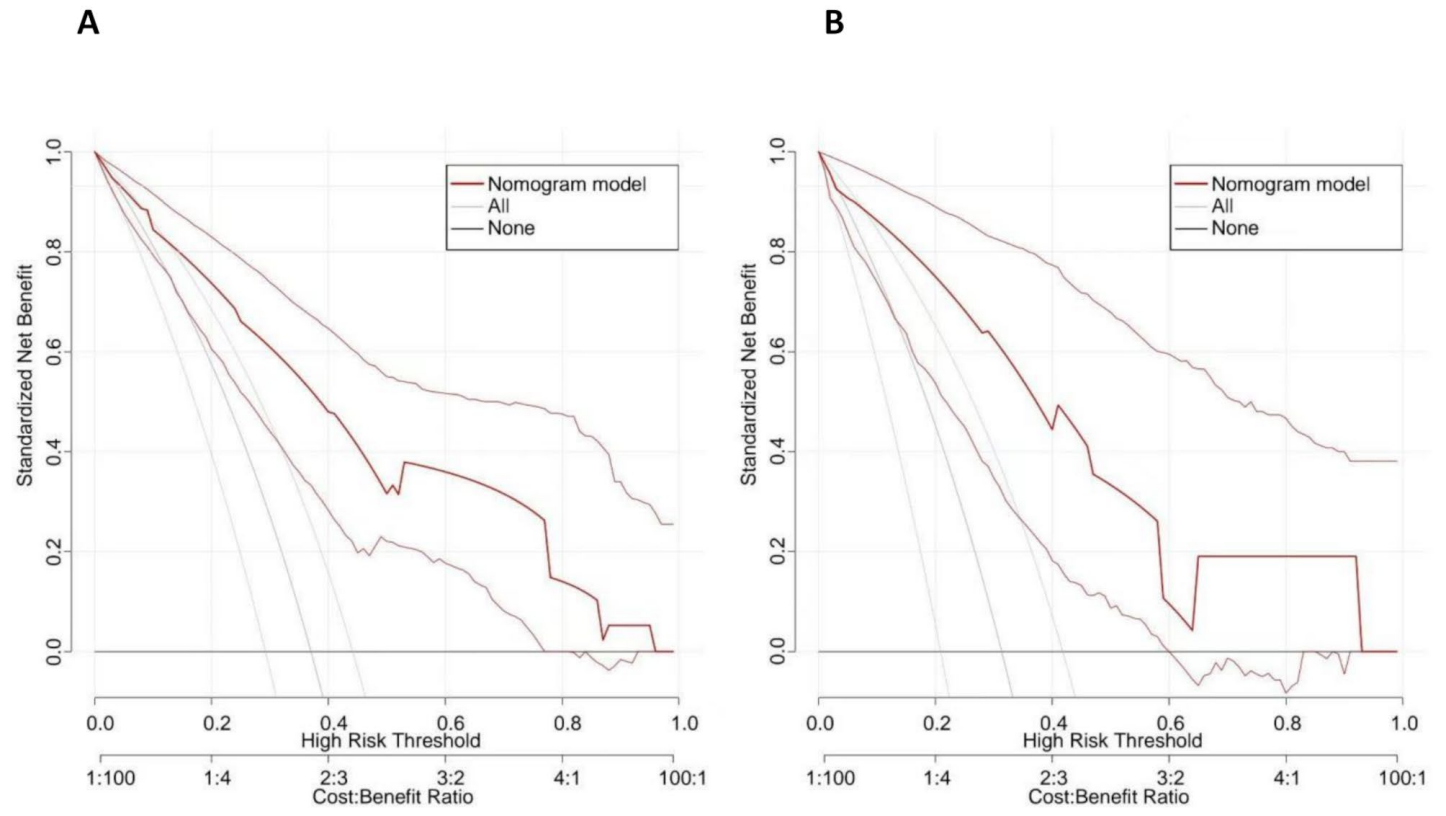
DCA curves for the training group and validation group. *A: DCA curves for the training group; **B**: DCA curves for the validation group

The DCA curves for the training group and the verification group are shown below (Fig. 4). The results showed that the model produced net benefits in both the training group and the validation group, which means that the prediction of CCLNM in clinical UPTC patients with a nomogram has good application value in clinical decision-making.

## Discussion

PTC demonstrates a propensity for early lymph node metastasis, with the central lymph node typically being the first area affected. Metastatic rates in this region have been reported to reach as high as 90% [9]. In patients diagnosed with UPTC of clinical N0 status, BCLNM is documented in up to 50% of patients. Moreover, an incidence rate of 6.7% has been recorded for ‘skip metastasis’ to the contralateral central lymph nodes [10]. In the present study, we demonstrated a CCLNM rate of 35.3% (78/221). Hence, for patients with UPTC, surgery confined to ipsilateral central lymph node dissection (ICLND) may not provide adequate coverage. Undetected positive lymph nodes during initial surgery could increase the tumor recurrence rate and complicate any subsequent surgeries. Although preventive BCLND can help us to accurately evaluate pathological staging, lymph nodes guide subsequent treatment but also increase the risk of postoperative parathyroid function disorder [11, 12]. Therefore, when delineating the suitable extent of lymph node dissection, surgeons must carefully consider strategies to minimize postoperative complications and tumor recurrence. This underscores the importance of rigorous preoperative and intraoperative evaluations to identify risk factors related to CCLNM in patients with UPTC, thereby defining a precise surgical roadmap.

In this study, we retrospectively examined the clinicopathological data of 221 patients and reported a CCLNM rate of 35.3% (78/221). Among these patients, 33.0% (73/221) had BCLNM, while 2.26% (5/221) had metastasis absent in the ipsilateral central lymph node but present in the contralateral central lymph node (skip metastasis). These findings are in line with the majority of the academic literature [6, 13]. Nonetheless, studies led by Roh and Sun et al. reported a lower CCLNM rate ranging between 3.88% and 30.63% [9, 14], which is in contrast with our recorded rate of 35.3%. This discrepancy could be attributed to two main factors: (1) our study did not include clinical lymph node (cN) data according to our enrollment criteria, and (2) our study had a relatively small sample size. With a high incidence of HT (26.9%) [15], cN assessment could be affected, leading to bias in our enrolled patients. Consequently, we did not confine our UPTC patient inclusion criteria to only cN0. Furthermore, by diligently excluding patients with isthmic thyroid tumors, we were able to enhance the reliability of our conclusions.

The prior literature has implicated several factors as independent risk factors for CCLNM in UPTC patients. These included male sex, age under 45 years, BMI exceeding 25, external thyroid invasion, tumor diameter greater than 1 cm, presence of vascular cancer thrombus, multifocal tumors, BRAF V-600E gene mutation, and ICLNM [9, 16–21]. Our multivariate analysis corroborated these findings, revealing male sex (*P* = 0.010, OR: 3.790, 95% CI: 1.373–10.465), external thyroid invasion (*P* = 0.040, OR: 6.364, 95% CI: 1.083–37.381), tumor diameter (*P* = 0.01, OR: 3.674, 95% CI: 1.372–9.839), and ICLNM (*P* < 0.001, OR: 38.552, 95% CI: 2.675–27.342) as independent predictors of CCLNM. In line with our findings, Kang SK’s study also integrated the number of ICLNMs as a variable and utilized a cutoff score of 5, demonstrating that while ICLNM served as an independent risk factor for CCLNM, there was no correlation with the number of ICLNMs [18]. Consequently, it may be beneficial to consider BCLND for UPTC patients exhibiting confirmed ICLNM. The focus should prioritize ICLNM presence over attempting to determine surgical scope by the quantity of ICLNM.

While PTC is more prevalent in the female population [1], it is markedly more aggressive in male patients, often manifesting as cervical lymph node metastasis [9, 22, 23]. Our results align with this observation, indicating that male patients diagnosed with UPTC tend to be at a greater risk of CCLNM.

Sun W et al., in their meta-analysis, suggested that a tumor diameter exceeding 2 cm serves as an independent risk factor for CCLNM in cN0 UPTC patients [9]. Consistent with most previous studies, our research also indicated that a tumor diameter greater than 1 cm is an independent predictor of CCLNM. However, the correlation between tumor diameter and CCLNM warrants further exploration. An investigation by Yan S suggested that BCLND may be an appropriate surgical approach for UPTC patients with a tumor diameter greater than 1 cm. On the other hand, it also increases the risk of short-term postoperative complications such as vocal cord paralysis and decreased parathyroid hormone (PTH) levels [6]. Hence, accurate preoperative evaluation of the surgical scope is indispensable for minimizing postoperative complications. We do not advocate prophylactic contralateral central lymph node dissection for UPTC patients with tumors less than 1 cm in diameter.

In a meta-analysis by Kim DH et al., it was found that the presence of ICLNM (odds ratio = 13.9118; 95% CI: 8.7096 ~ 22.2213) showed the strongest correlation with CCLNM in UPTC patients [22], a finding that aligns with the conclusions drawn in this paper. Consequently, it is recommended that UPTC patients with confirmed ICLNM through preoperative US-FNAB or intraoperative frozen pathology undergo BCLND.

Moreover, the study revealed a heightened risk of cervical lymph node metastasis in patients where the tumor infiltrates the surrounding thyroid tissue, with ETE emerging as an independent risk factor for CCLNM in UPTC patients. These results are in line with the findings of Feng JW and Kim DH et al. [21][23]. Currently, preoperative ultrasonography has demonstrated significant predictive value in assessing the extrathyroidal invasion of thyroid tumors, and the widespread use of intraoperative frozen pathological examination can serve as a valuable tool for determining the appropriate surgical scope. Furthermore, ETE is indicative of the aggressive nature of PTC and plays a pivotal role in predicting patient prognosis. Upon the occurrence of ETE, the risk of tumor recurrence and mortality escalates [24].

Due to the inclusion of a multicenter sample in this study, uniform testing for BRAF V-600E gene mutations could not be conducted and hence was not included in the analysis. Furthermore, discrepancies with previous studies in factors such as age, multifocality, HT, vascular cancer thrombus, and BMI are likely attributable to the limitations posed by the small sample size.

In essence, this retrospective study comprehensively analyzed clinicopathological data from 221 samples collected across multiple centers to develop a predictive model for determining CCLNM in UPTC patients based on four distinct risk factors: sex, tumor diameter, external thyroid invasion, and ICLNM. The resulting model demonstrated robust differentiation and calibration, yielding tangible clinical benefits for decision-making processes. By integrating the nomogram with preoperative and intraoperative assessment parameters such as male sex, tumor diameter > 1 cm, external thyroid invasion, and ICLNM in UPTC patients, personalized and precise surgical delineation can be achieved, optimizing treatment strategies. However, it is important to note the limitations of this study, which include its relatively small sample size and the absence of external validation. Therefore, there is a clear imperative to bolster data supplementation to enhance the generalizability and applicability of the prediction model.

## Electronic supplementary material

Below is the link to the electronic supplementary material.

Supplementary Material 1

## Data Availability

The raw data supporting the conclusions of this article will be made available by the authors, without undue reservation.

## References

[1] Haugen BR, Alexander EK, Bible KC, Doherty GM, Mandel SJ, Nikiforov YE, Pacini F, Randolph GW, Sawka AM, Schlumberger M, Schuff KG, Sherman SI, Sosa JA, Steward DL, Tuttle RM, Wartofsky L. 2015 American Thyroid Association Management Guidelines for adult patients with thyroid nodules and differentiated thyroid Cancer: the American Thyroid Association Guidelines Task Force on thyroid nodules and differentiated thyroid Cancer. Thyroid. 2016;26(1):1–133. 10.1089/thy.2015.0020. PMID: 26462967; PMCID: PMC4739132. 26462967 10.1089/thy.2015.0020 PMC4739132

[2] Yan B, Hou Y, Chen D, He J, Jiang Y. Risk factors for contralateral central lymph node metastasis in unilateral cN0 papillary thyroid carcinoma: a meta-analysis. Int J Surg. 2018;59:90–8. Epub 2018 Oct 17. PMID: 30。. 30342280 10.1016/j.ijsu.2018.09.004

[3] Jianyong L, Jinjing Z, Zhihui L, Tao W, Rixiang G, Jingqiang Z. A Nomogram Based on the Characteristics of Metastatic Lymph Nodes to Predict Papillary Thyroid Carcinoma Recurrence. Thyroid. 2018;28(3):301–310. 10.1089/thy.2017.0422. PMID: 29439612. 10.1089/thy.2017.0422 29439612

[4] Machens A, Hinze R, Thomusch O, Dralle H. Pattern of nodal metastasis for primary and reoperative thyroid cancer. World J Surg. 2002;26(1):22–8. 10.1007/s00268-001-0176-3. Epub 2001 Nov 22. PMID: 11898029. 11898029 10.1007/s00268-001-0176-3

[5] Liu H, Li Y, Mao Y. Local lymph node recurrence after central neck dissection in papillary thyroid cancers: A meta analysis. Eur Ann Otorhinolaryngol Head Neck Dis. 2019;136(6):481–487. 10.1016/j.anorl.2018.07.010. Epub 2019 Jun 10. PMID: 31196. 10.1016/j.anorl.2018.07.010 31196800

[6] Yan S, Yu J, Zhao W, Wang B, Zhang L. Prophylactic bilateral central neck dissection should be evaluated based on prospective randomized study of 581 PTC patients. BMC Endocr Disord. 2022;22(1):5. 10.1186/s12902-021-00909-0. PMID: 34983475; PMCID: PMC8725302. 34983475 10.1186/s12902-021-00909-0 PMC8725302

[7] Guang Y, He W, Zhang W, Zhang H, Zhang Y, Wan F. Clinical study of Ultrasonographic Risk Factors for Central Lymph Node Metastasis of Papillary thyroid carcinoma. Front Endocrinol (Lausanne). 2021;12:791970. 10.3389/fendo.2021.791970. PMID: 34917039; PMCID: PMC8669800. 34917039 10.3389/fendo.2021.791970 PMC8669800

[8] Mulla M, Schulte KM. Central cervical lymph node metastases in papillary thyroid cancer: a systematic review of imaging-guided and prophylactic removal of the central compartment. Clin Endocrinol (Oxf). 2012;76(1):131-6. 10.1111/j.1365-2265.2011.04162.x. PMID: 21722150. 10.1111/j.1365-2265.2011.04162.x 21722150

[9] Sun W, Zheng B, Wang Z, Dong W, Qin Y, Zhang H. Meta-analysis of risk factors for CCLNM in patients with unilateral cN0 PTC. Endocr Connect. 2020;9(5):387–95. 10.1530/EC-20-0058. PMID: 32272445; PMCID: PMC7219143. 32272445 10.1530/EC-20-0058 PMC7219143

[10] Koo BS, Choi EC, Yoon YH, Kim DH, Kim EH, Lim YC. Predictive factors for ipsilateral or contralateral central lymph node metastasis in unilateral papillary thyroid carcinoma. Ann Surg. 2009;249(5):840-4. 10.1097/SLA.0b013e3181a40919. PMID: 19387316. 10.1097/SLA.0b013e3181a40919 19387316

[11] Giordano D, Valcavi R, Thompson GB, Pedroni C, Renna L, Gradoni P, Barbieri V. Complications of central neck dissection in patients with papillary thyroid carcinoma: results of a study on 1087 patients and review of the literature. Thyroid. 2012;22(9):911–7. 10.1089/thy.2012.0011. Epub 2012 Jul 24. PMID: 22827494. 22827494 10.1089/thy.2012.0011

[12] Giordano D, Frasoldati A, Gabrielli E, Pernice C, Zini M, Castellucci A, Piana S, Ciarrocchi A, Cavuto S, Barbieri V. Long-term outcomes of central neck dissection for cN0 papillary thyroid carcinoma. Am J Otolaryngol 2017 Sep-Oct;38(5):576–81. doi: 10.1016/j.amjoto.2017.06.004. Epub 2017 Jun 14. PMID: 28599790. 10.1016/j.amjoto.2017.06.004 28599790

[13] Ito Y, Jikuzono T, Higashiyama T, Asahi S, Tomoda C, Takamura Y, Miya A, Kobayashi K, Matsuzuka F, Kuma K, Miyauchi A. Clinical significance of lymph node metastasis of thyroid papillary carcinoma located in one lobe. World J Surg. 2006;30(10):1821-8. 10.1007/s00268-006-0211-5. PMID: 16983469. 10.1007/s00268-006-0211-5 16983469

[14] Roh JL, Kim JM, Park CI. Central lymph node metastasis of unilateral papillary thyroid carcinoma: patterns and factors predictive of nodal metastasis, morbidity, and recurrence. Ann Surg Oncol. 2011;18(8):2245–50. 10.1245/s10434-011-1600-z. Epub 2011 Feb 15. PMID: 21327454. 21327454 10.1245/s10434-011-1600-z

[15] Chen WMD, Li ZMD, Zhu JPD, Lei JPD, Wei T. MD∗. Unilateral papillary thyroid carcinoma treated with contralateral central lymph node dissection: A nomogram to aid in decision-making. Medicine 99(38):p e22200, September 18, 2020. | 10.1097/MD.0000000000022200 10.1097/MD.0000000000022200 PMC7505319 32957351

[16] Xia BY, Abuduwaili M, Fei Y, Xing ZC, Liu Y, Zhang LY, Su AP, Zhu JQ. [Analysis of correlation factors of contralateral central lymph node metastasis in unilateral papillary thyroid carcinoma with lateral cervical lymph node metastasis]. Zhonghua Wai Ke Za Zhi. 2021;59(6):502–506. Chinese. 10.3760/cma.j.cn112139-20200706-00541. PMID: 34102735. 10.3760/cma.j.cn112139-20200706-00541 34102735

[17] Tan HL, Huang BQ, Li GY, Wei B, Chen P, Hu HY, Liu M, Ou-Yang DJ, Yang Q, Qin ZE, Shi QM, Li N, Huang P, Chang S. A prediction model for Contralateral Central Neck Lymph Node Metastases in unilateral papillary thyroid Cancer. Int J Endocrinol. 2021;2021:6621067. 10.1155/2021/6621067. PMID: 34306071; PMCID: PMC8263281. 34306071 10.1155/2021/6621067 PMC8263281

[18] Kang SK, Kim DI, Im DW, Lee S, Choi JB, Jung YJ, Kim HY. A retrospective study of factors affecting contralateral central-neck lymph node metastasis in unilateral papillary thyroid carcinoma. Asian J Surg. 2023;46(9):3485–90. Epub 2022 Nov 10. PMID: 36372709. 36372709 10.1016/j.asjsur.2022.10.081

[19] Zhou B, Qin J. High-risk factors for lymph node metastasis in contralateral central compartment in unilateral papillary thyroid carcinoma(cT1N0). Eur J Surg Oncol. 2021;47(4):882–7. 10.1016/j.ejso.2020.10.018. Epub 2020 Oct 18. PMID: 33092967. 33092967 10.1016/j.ejso.2020.10.018

[20] Feng JW, Qu Z, Ye J, Hong LZ, Liu SY, Wang F, Jiang Y, Hu J. Nomograms to predict ipsilateral and contralateral central lymph node metastasis in clinically lymph node-negative patients with solitary isthmic classic papillary thyroid carcinoma. Surgery. 2021;170(6):1670–9. Epub 2021 Jul 16. PMID: 34275617. 34275617 10.1016/j.surg.2021.06.027

[21] Shukla N, Osazuwa-Peters N, Megwalu UC. Association between Age and Nodal Metastasis in papillary thyroid carcinoma. Otolaryngol Head Neck Surg. 2021;165(1):43–9. 10.1177/0194599820966995. Epub 2020 Oct 20. PMID: 33076796. 33076796 10.1177/0194599820966995

[22] Kim DH, Kim GJ, Kim SW, Hwang SH. Predictive value of ipsilateral central lymph node metastasis for contralateral central lymph node metastasis in patients with thyroid cancer: systematic review and meta-analysis. Head Neck. 2021;43(10):3177–84. 10.1002/hed.26787. Epub 2021 Jun 14. PMID: 34124791. 34124791 10.1002/hed.26787

[23] Mao J, Zhang Q, Zhang H, Zheng K, Wang R, Wang G. Risk factors for Lymph Node Metastasis in Papillary thyroid carcinoma: a systematic review and Meta-analysis. Front Endocrinol (Lausanne). 2020;11:265. 10.3389/fendo.2020.00265. PMID: 32477264; PMCID: PMC7242632. 32477264 10.3389/fendo.2020.00265 PMC7242632

[24] Lin JD, Hsueh C, Chao TC. Soft tissue invasion of papillary thyroid carcinoma. Clin Exp Metastasis. 2016;33(6):601–8. 10.1007/s10585-016-9800-3. Epub 2016 May 6. PMID: 27154220; PMCID: PMC4947096. 27154220 10.1007/s10585-016-9800-3 PMC4947096

